# Spectral Correlates of Encoding Distinguish Good from Poor Learners

**DOI:** 10.64898/2026.07.29.741612

**Published:** 2026-08-04

**Authors:** Ben Falkenburg, Adam Broitman, Michael J. Kahana

## Abstract

Individuals vary in their ability to encode and recall new experiences, yet neural markers of memory have rarely been linked to these trait-level differences. Subsequent memory effects (SMEs) identify patterns of neural activity during encoding that predict whether an item will later be remembered or forgotten. In scalp EEG, SMEs are typically characterized by increased theta and gamma power with decreased alpha, a profile that has been interpreted as a signature of effective encoding. Here, we tested whether SMEs reflect individual differences in mnemonic ability by analyzing over 1.3 million encoding events from a large free recall dataset. At the group level, alpha suppression and theta enhancement predicted successful encoding, while gamma did not predict subsequent memory after controlling for serial position. Across individuals, better learners showed reduced SMEs in all three frequency bands. The neural contrast that defines successful encoding was therefore most pronounced among the poorest learners. These findings suggest that high-performing individuals are less reliant on phasic spectral activity for successful encoding, and that SMEs capture stable individual differences in encoding efficiency.

**Highlights:**

- EEG activity during learning differentiates better from poorer learners
- EEG markers of successful encoding also reflect stable learning ability
- Higher-performing individuals show attenuated theta–alpha–gamma signatures
- EEG dynamics track both transient states and trait-like ability differences

## Full Text Availability

The license terms selected by the author(s) for this preprint version do not permit archiving in PMC. The full text is available from the preprint server.

